# Complete Genome Sequence of *Mycoplasma putrefaciens* Strain 9231, One of the Agents of Contagious Agalactia in Goats

**DOI:** 10.1128/genomeA.00354-13

**Published:** 2013-06-13

**Authors:** Virginie Dupuy, Pascal Sirand-Pugnet, Eric Baranowski, Aurélien Barré, Marc Breton, Carole Couture, Emilie Dordet-Frisoni, Patrice Gaurivaud, Daniel Jacob, Claire Lemaitre, Lucía Manso-Silván, Macha Nikolski, Laurent-Xavier Nouvel, François Poumarat, Florence Tardy, Patricia Thébault, Sébastien Theil, Christine Citti, Alain Blanchard, François Thiaucourt

**Affiliations:** CIRAD, UMR CMAEE, Montpellier, Francea; INRA, UMR1309 CMAEE, Montpellier, Franceb; INRA, UMR 1332 de Biologie du Fruit et Pathologie, Villenave d’Ornon, Francec; Univ. Bordeaux, UMR 1332 de Biologie du Fruit et Pathologie, Villenave d’Ornon, Franced; INRA, UMR 1225, IHAP, Toulouse, Francee; Université de Toulouse, INP, ENVT, UMR1225, IHAP, Toulouse, Francef; Université de Bordeaux, Centre de Bioinformatique et de Génomique Fonctionnelle Bordeaux, CBiB, Bordeaux, Franceg; Anses, Laboratoire de Lyon, UMR Mycoplasmoses des Ruminants, Lyon, Franceh; Université de Lyon, VetAgro Sup, UMR Mycoplasmoses des Ruminants, Marcy L’Etoile, Francei; Université Bordeaux, LaBRI, UMR 5800, Talence, Francej

## Abstract

*Mycoplasma putrefaciens* is one of the etiologic agents of contagious agalactia in goats. We report herein the complete genome sequence of *Mycoplasma putrefaciens* strain 9231.

## GENOME ANNOUNCEMENT

*Mycoplasma putrefaciens* is one of the etiologic agents of contagious agalactia in goats, a syndrome listed by the World Organisation for Animal Health (OIE) (1) and characterized by mastitis, arthritis, keratoconjunctivitis, pneumonia, and septicemia (“MAKePS”) (2). It can also be found as a commensal organism in the ear canal (3). This mycoplasma is phylogenetically related to the *Mycoplasma mycoides* cluster, which comprises five major ruminant pathogens: *M. mycoides* subsp. *mycoides*, *M. mycoides* subsp. *capri*, *M. capricolum* subsp. *capricolum*, *M. capricolum* subsp. *capripneumoniae*, and *M. leachii* (4, 5). *M. putrefaciens* was characterized only in 1976 (6). This species is not as frequently isolated as some members of the *Mycoplasma mycoides* cluster (7), although it has a worldwide distribution (3, 8, 9). Since the whole annotated genome of the type strain KS1^T^ has been recently published (10), an additional *M. putrefaciens* genome will allow comparative analyses to gain insight into the pan genome of the species as well as the intra-species polymorphism.

The genome sequence was determined by the Sanger method (GATC, Constanz, Germany) and resulted in 8.4× average depth. *De novo* assembly was performed using SeqMan (version 7.0; DNASTAR Lasergene, Madison, WI) and resulted in 3 scaffolds. Gap closure was completed by PCR amplification and Sanger sequencing. Annotation was conducted using a customized version of the CAAT-BOX platform (PMID, 14752000) with automatic preannotation for coding sequences (CDS), followed by expert validation as previously described (11).

The complete genome consisted of an 859,996-bp sequence with a global G+C content of 26.96%. It is composed of a single circular chromosome that contains 749 genes, of which 667 correspond to CDS, 44 to pseudogenes, and 38 to structural RNAs. Unlike the genome of strain KS1^T^, in which no functional mobile elements were found, a 23.9-kb-long integrative conjugative element (ICE) and a single copy of an IS3 family insertion sequence were identified in 9231, which mainly explains their difference in size. The ICE comprises 16 CDS, including the *traG* and *traE* genes. The last two ICE genes are duplicated in the genome and may represent a vestige of a previous ICE integration.

The availability of high-quality genome sequences, as for *M. putrefaciens* 9231, and comparative analyses with related species will improve our understanding of the pathogenomic evolution of ruminant pathogens.

### Nucleotide sequence accession number.

The annotated genome sequence was deposited in GenBank under accession no. CP004357 and in the MolliGen database.
